# Capability of tip-growing plant cells to penetrate into extremely narrow gaps

**DOI:** 10.1038/s41598-017-01610-w

**Published:** 2017-05-03

**Authors:** Naoki Yanagisawa, Nagisa Sugimoto, Hideyuki Arata, Tetsuya Higashiyama, Yoshikatsu Sato

**Affiliations:** 1 Division of Biological Science, Graduate School of Science, Nagoya University, Furo-cho, Chikusa-ku, Nagoya Aichi, 464-8602 Japan; 2 JST ERATO Higashiyama Live-Holonics Project, Nagoya University, Furo-cho, Chikusa-ku, Nagoya, Aichi, 464-8601 Japan; 3 Institute of Transformative Bio-Molecules (ITbM), Nagoya University, Furo-cho, Chikusa-ku, Nagoya, Aichi, 464-8601 Japan; 4 Institute of Industrial Science (IIS), The University of Tokyo, Komaba, Meguro-ku, Tokyo, 153-8505 Japan

## Abstract

Plant cells are covered with rigid cell walls, yet tip-growing cells can elongate by providing new cell wall material to their apical regions. Studies of the mechanical properties of tip-growing plant cells typically involve measurement of the turgor pressure and stiffness of the cells’ apical regions. These experiments, however, do not address how living tip-growing cells react when they encounter physical obstacles that are not substantially altered by turgor pressure. To investigate this issue, we constructed microfabricated platforms with a series of artificial gaps as small as 1 μm, and examined the capability of tip-growing plant cells, including pollen tubes, root hairs, and moss protonemata, to penetrate into these gaps. The cells were grown inside microfluidic chambers and guided towards the gaps using microdevices customized for each cell type. All types of tip-growing cells could grow through the microgaps with their organelles intact, even though the gaps were much smaller than the cylindrical cell diameter. Our findings reveal the dramatic physiological and developmental flexibility of tip-growing plant cells. The microfluidic platforms designed in this study provide novel tools for the elucidation of the mechanical properties of tip-growing plant cells in extremely small spaces.

## Introduction

Unlike diffuse-growing cells, which exhibit overall growth, tip-growing cells elongate in a limited region of the cells’ apices. The molecular processes that regulate apical cell growth have not yet been conclusively deciphered^1, 2^, but various studies have confirmed that maintaining the proper elongation of tip-growing cells requires exquisite coordination between hydrostatic turgor pressure and stiffness of the apical cell wall^3–5^. Several techniques are currently available to measure these properties of plant cells. For instance, the incipient plasmolysis method^6^ is often employed to measure turgor pressure in the pollen tube (PT), and cellular force microscopy (CFM) can be used to measure the stiffness of tissues and cells in a non-invasive manner^7, 8^. CFMs are equipped with a sensor probe that directly indents the cellular surface, and its displacement is used to quantify cell wall stiffness. Such technological advances in the field of Micro Electro Mechanical Systems (MEMS) allow us to measure physical quantities at the single-cell level. Likewise, microfluidic devices originating from MEMS-based technology have also received considerable attention from the plant science community^9–11^; recently, the penetrative force of tip-growing PTs was successfully measured using a polydimethylsiloxane (PDMS) microfluidic device by taking advantage of the elastic properties of PDMS^12^. These quantitative methods for measuring turgor pressure and stiffness in the apical cell wall are indispensable, especially for the investigation of the mechanical properties of tip-growing cells.

Nonetheless, the ability of tip-growing cells to overcome a series of physical barriers *in vivo* cannot be elucidated from these two measurements alone. To predict how these cells would react when faced with a confined structure, it is also important to understand the deformation capacity of the cell, as well as continuous elongation, under such restrictive conditions. For example, PTs are thought to be subjected to this type of compressive stress when they invade a pistil and penetrate the micropyle to deliver sperm cells (SCs) for fertilization. In the current study, to replicate such conditions *in vitro*, we fabricated a series of firm artificial gaps as narrow as 1 µm on microfluidic platforms made of PDMS and investigated the ability of tip-growing plant cells to overcome these physical barriers. We utilized this system to examine three types of tip-growing plant cells: PTs, root hairs, and moss protonemata.

## Results

### PTs can deform their apical shapes to pass through microgaps

**Figure 1:**
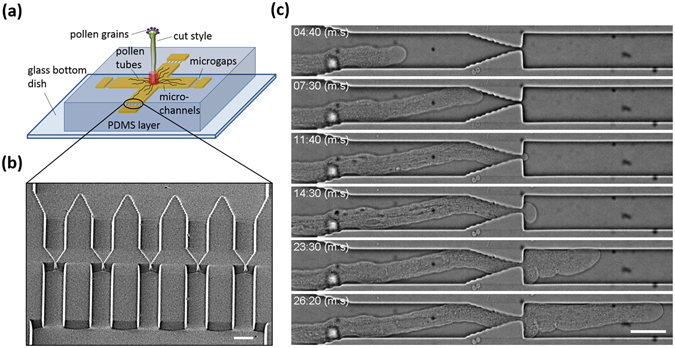
*In vitro* culture of *T. fournieri* pollen tubes (PTs) in the microfluidic platform. (**a**) Schematic drawing of the microdevice used for the PT study. A cut *T. fournieri* style is inserted into an access hole to the fluidic channels located at the center of the device. The PTs growing in the style eventually enter the microchannels, which are filled with PT growth medium. The microgaps are located 3.7 mm from the access hole. (**b**) Scanning electron microscopy (SEM) image of microchannels with 1-μm-wide gaps (4 μm in height). Scale bar, 20 µm. (**c**) Time-lapse images of PT elongation through a 1-μm gap. See Supplementary Movie 1. Scale bar, 20 µm.

Figure 1a shows a schematic drawing of the microdevice used to study the ability of a PT to pass through an extremely narrow gap. After manually transferring pollen grains to a *Torenia fournieri* stigma, the cut style was placed in the inlet of the device. Approximately 5–6 hours after pollination, PTs emerged from the end of the cut style and continuously grew inside the microchannels, which were filled with growth medium. While the PTs initially grew in random orientations in the microfluidic chamber, we subsequently separated the PTs and guided each one toward a microgap (Supplementary Fig. S1), employing the channel structure shown in Fig. 1b. The channels comprised 1-μm, 2-μm, and 3-μm wide gaps at a fixed height (4 μm). We captured time-lapse images of PT elongation at the gap region and found that the PT became constricted as it grew toward the microgap, eventually penetrating it completely (Fig. 1c, Supplementary Movie 1). In some cases, the gap became somewhat wider as the PT crossed it, suggesting that the turgor pressure was strong enough to deform the shape of the PDMS structure (Supplementary Fig. S2); nevertheless, the width of the gap was still much smaller than the diameter of the PT (~8 μm). A total of 81% (*n* = 52) of PTs successfully crossed the 1-μm gap and continued to grow without bursting (Supplementary Fig. S3). The amount of time required for the PTs to penetrate through the gap varied: 53% of PTs crossed the gap within 30 min, whereas 23% crossed the gap up to 1 h. The remaining 24% of PTs struggled to cross the gap, taking approximately 1–2 h to complete this process. We also found that the tip-growth rates were comparable between before (22 ± 4 s.d. µm·min^−1^) and after (23 ± 3 s.d. µm·min^−1^) crossing the gap (P = 0.37, two-sided paired t-test, *n* = 12, Supplementary Fig. S4).

Although most PTs were able to penetrate the 1-μm gap, this was not the case when the length of the gap was extended. We also prepared longer gap channels (50, 100, and 200 μm) with a fixed width and height (1 μm and 4 μm, respectively) and investigated whether the PTs were still capable of penetrating through these longer gaps (Supplementary Fig. S5). For the 50-μm channel, only 21% (*n* = 33) of PTs passed through the gap completely, whereas the remaining PTs became stuck or burst in the middle of the channel. For the 200-μm channel, the penetration rate further dropped to 3% (*n* = 29), indicating that most PTs cannot maintain tip growth over a long distance.

### The vegetative nucleus and SCs can penetrate a PDMS microgap

**Figure 2:**
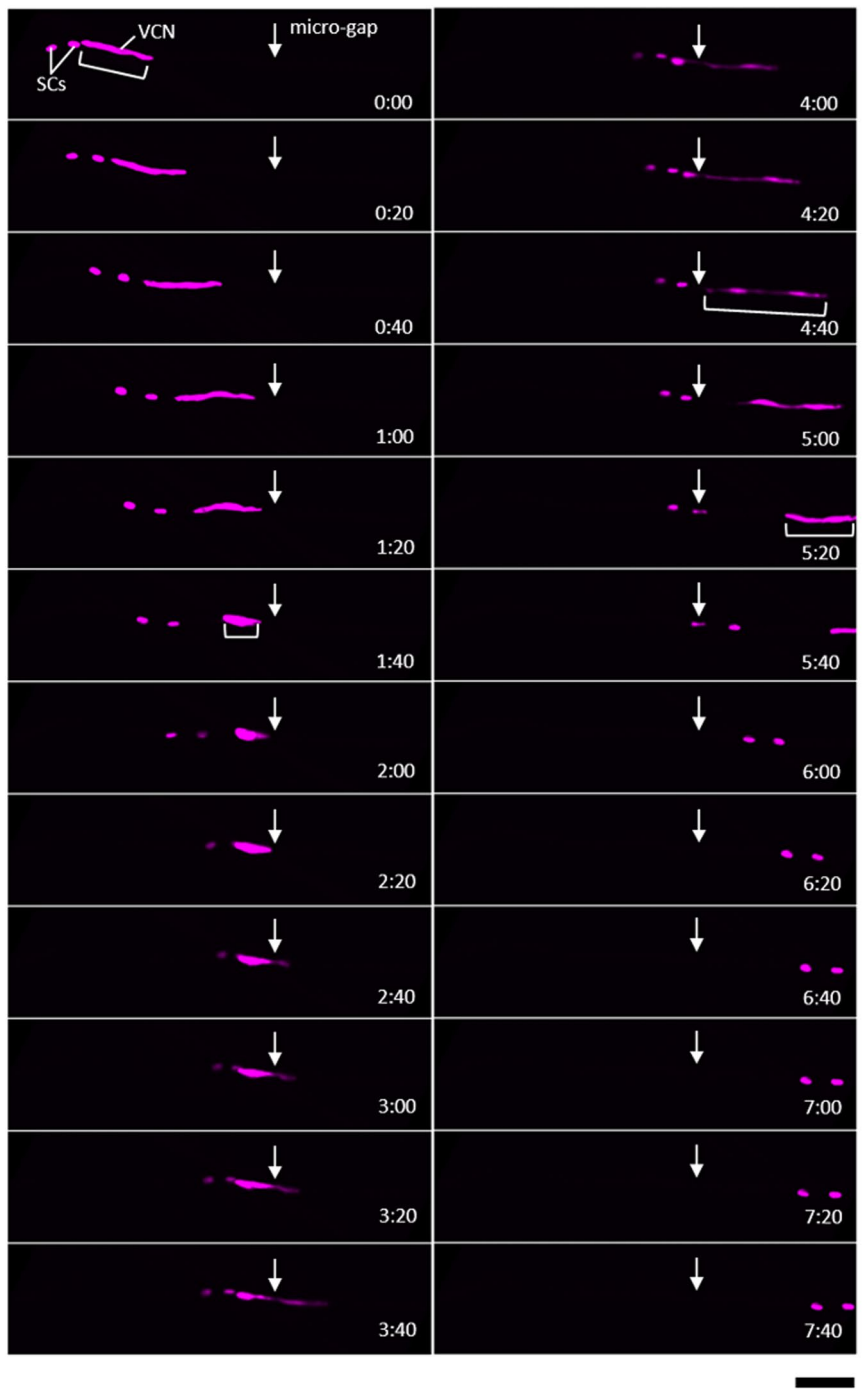
Vegetative nucleus and sperm cells of *T. fournieri* passing through a 1-µm wide gap. Fluorescently labeled vegetative nuclei (VN) and sperm cell (SC) nuclei are visualized in the *RPS5Ap*::*H2B-tdTomato* line. See Supplementary Movie 2. Timestamp, min:sec. Scale bars, 50 µm.

During angiosperm pollen development, generative and vegetative cells are produced as a result of asymmetric cell division^13^. Subsequently, the second mitotic division of a generative cell leads to the production of two SCs in the cytoplasm of the vegetative cell. While the roles of the vegetative-cell nucleus (VN) in PTs remain largely unknown, the VN is normally found near the apical region and likely plays a vital role in regulating PT growth^14, 15^. To determine whether the VN can also penetrate through a 1-μm gap, we performed fluorescent time-lapse observations using a *RPS5Ap*::*H2B-tdTomato* line^16^, in which the VN and SC nuclei are labeled (Fig. 2, Supplementary Movie 2). The VN initially had a rod-like shape but became rounded when it encountered the gap. Also, the anterior SC collided with the VN at the gap, although it typically remained distant during tip growth. The VN gradually entered into the gap and stretched out as it passed through this region. After the VN had completely crossed the gap, it returned to its initial rod-like shape and migrated toward the apex of the PT. The change in VN shape during the gap penetration process was a common phenomenon among all the PTs (*n* = 7). Moreover, two SCs in the PT became constricted and penetrated through the gap without any visually notable damage (Fig. 2, time 5:20 and 5:40). We examined the penetration capability of the VN and SCs through the 1-μm gap and found that all (*n* = 7) were able to cross the gap as long as the PT had successfully passed through it.

### Root hairs can deform their apical shapes to penetrate microgaps

A root hair is a single tip-growing plant cell that emerges from epidermal cells in the root. The large surface area of the root hair is thought to help roots absorb adequate water and nutrients from their surroundings, which is essential for plant growth^17^. A recent study showed that root hairs also play an important role in root anchorage and soil penetration^17^; hence, the capability of root hairs to penetrate through the small space such as packed soil particles likely influences plant growth.

**Figure 3:**
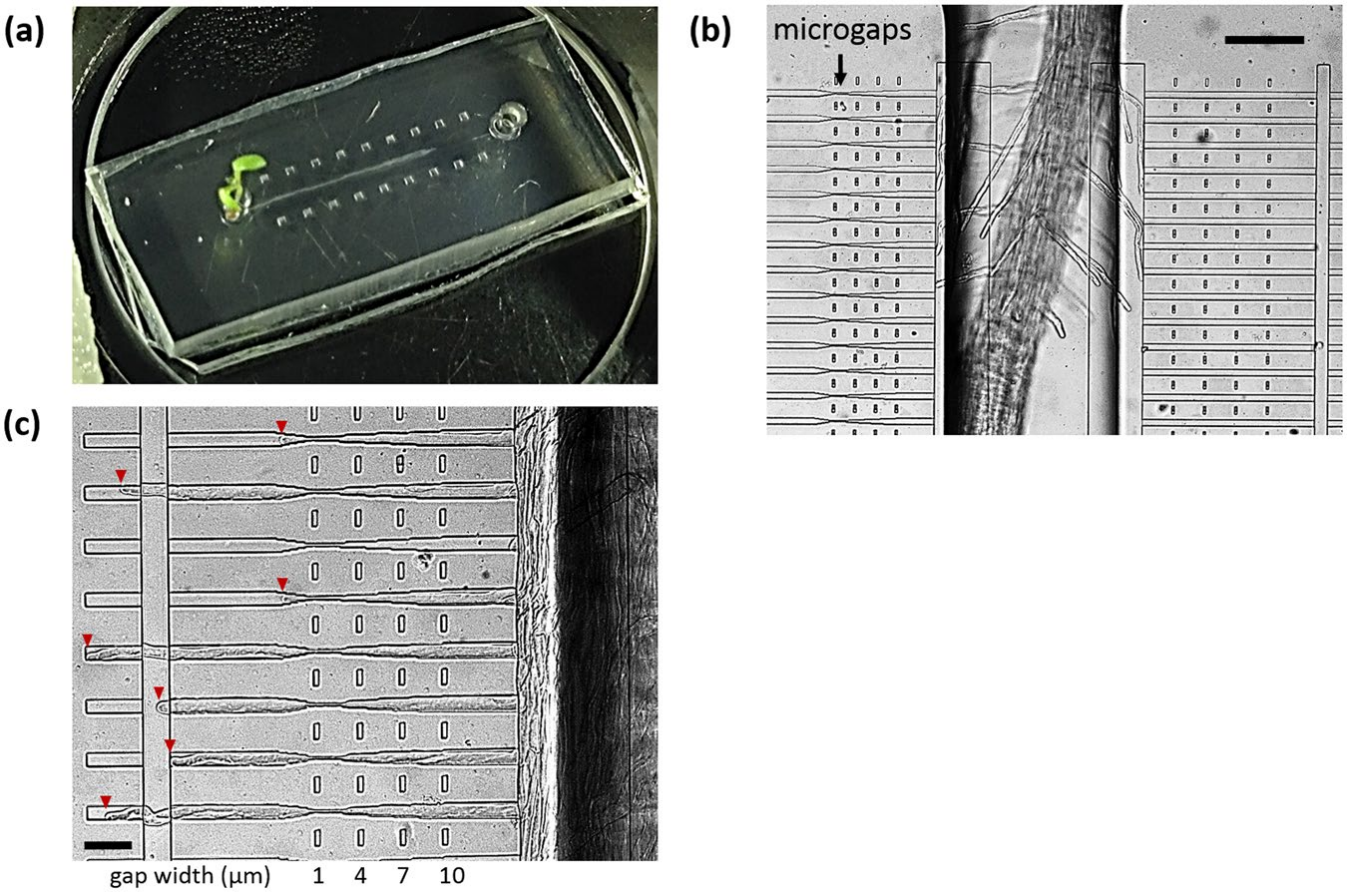
*In vitro* culture of *A. thaliana* root hairs in the microfluidic platform. (**a**) Microfluidic platform used to test the capability of root hairs to penetrate through microgaps. (**b**) Representative image of root and root hair elongation in this platform. Microgaps are located on the left side of the root growth chamber. Scale bar, 100 µm. (**c**) Representative image of root hairs penetrating through 1-µm gaps. Scale bar, 30 µm.

The microdevice used in this study is shown in Fig. 3a. We initially filled all of the microchannels with growth medium for *Arabidopsis thaliana* and placed an Arabidopsis seed in the hole connected to the channels. After the seed had germinated, the root started growing through the deep channel (200 μm in height) located in the center (Fig. 3b), while the leaves developed outside of the device. The shallow channels (4 μm in height) were perpendicularly connected to the root growth chamber. On the left side, the channel entrances were 10 μm wide and gradually narrowed to 1 μm. On the right side, the width of the channels was fixed at 10 μm, which is wider than the diameter of a root hair (~7 μm). Figure 3c shows a representative image of root hair growth in the narrowing channels. Unlike PT elongation, root hair elongation was rather limited; some root hairs stopped elongating in the middle of the 10-µm control channels, even though cytoplasmic streaming was still observed, resulting in underestimation of successful penetration of the microgap. However, 77% of the root hairs (*n* = 61) that entered the narrowing channels passed through the 1-μm gap. This suggested that the shape of the root hair apex can be deformed according to its surroundings, enabling it to penetrate gaps much smaller than the diameter of its apical region.

We also examined the location of the nucleus in root hairs that had crossed the microgap using a *UBQ10pro*::*H2B-mClover* line, in which the nuclei in roots and root hairs are labeled. Fluorescent root hair nuclei were observed to have passed through the 1-μm gap as well (Supplementary Fig. S6).

### Moss protonemata can be deformed to grow in the narrow microchannels

The gametophytic tissue in the moss *Physcomitrella patens* has long been studied as a model system to investigate tip growth in plants^18^. The lifecycle of the moss begins with spore germination via apical growth, followed by cell division. The apical cells repeatedly divide to form a thread-like tissue known as the protonema; thus, unlike other tip-growing plant cells such as PTs and root hairs, the protonema is a multicellular tissue. The proper elongation of the protonema cell in moss is therefore directly linked to the establishment of the plant body, a process critical for propagation. Tip growth in moss protonemata has traditionally been studied using an agar layer composed of growth medium, but a recent study showed that a PDMS microfluidic device can also be used as a growth chamber^19^.

**Figure 4:**
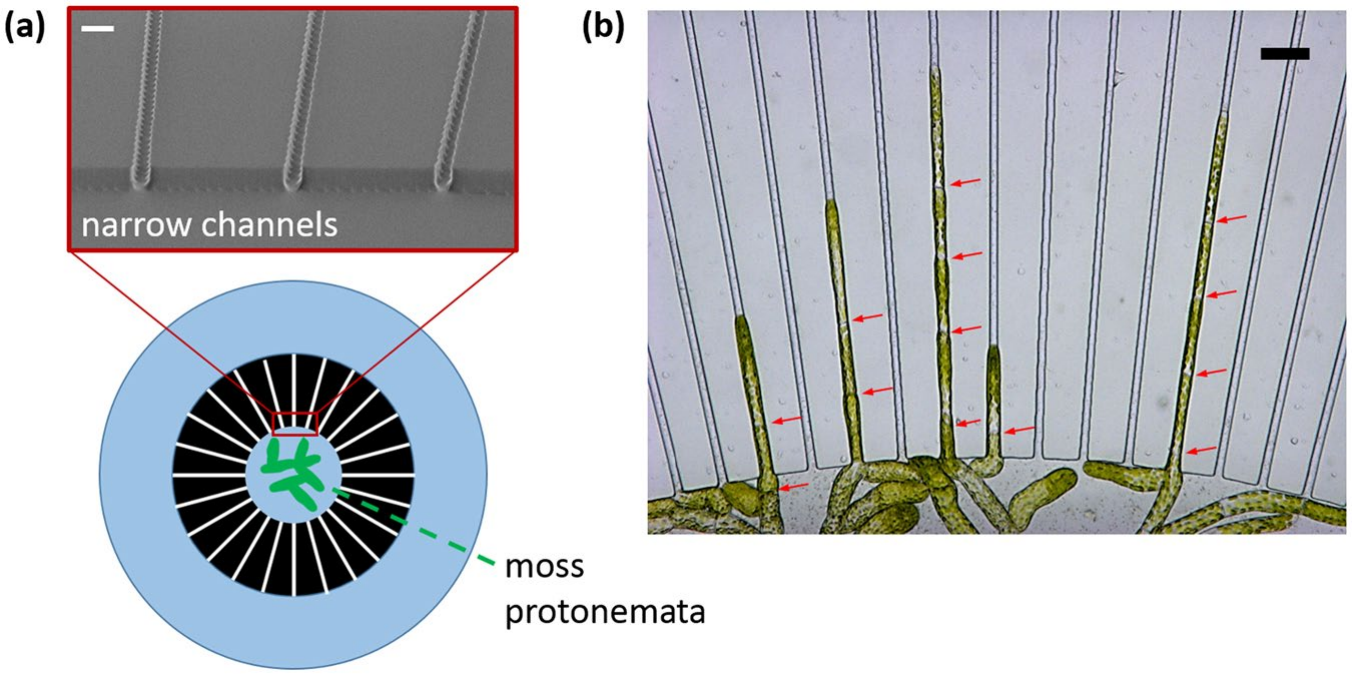
*In vitro* culture of moss (*P. patens*) protonemata in a microfluidic platform. (**a**) Schematic microchannel design and SEM image of the narrow channels used for the moss protonema penetration study. Scale bar, 10 µm. (**b**) Representative image of the elongation of moss protonemata through the narrow channels. The positions of septa are indicated by arrows. Scale bar, 30 µm.

In the current study, we examined the penetration capability of apical cells of protonemata through physically confined spaces. Our microdevice for the moss protonemata growth consisted of a series of narrow channels (width: 5 µm, height: 10 µm) that were equally distributed around the sample inlet (Fig. 4a). The height at the center of the growth chamber was also 10 µm, which is approximately half the diameter of an apical protonema cell (18–20 µm), to allow only a small fraction of the protonemata in the sample inlet to enter the device. After incubating the protonemata for 7–9 days, some protonemata entered the channels and continued to grow (Fig. 4b). Unexpectedly, in addition to elongation of the apical cells, consecutive cell divisions were also observed in this tight space. Although the narrow channels widened somewhat as the protonema cells grew inside them, due to increased cellular turgor pressure, the cross-sectional area of the channels was still much smaller than the typical diameter of a protonema cell apex.

Next, we investigated the limitations of protonema cell growth by further reducing the size of the confined space. For this purpose, we utilized two types of devices (Fig. 1a and Supplementary Fig. S5a), which were originally prepared to test the penetration capability of PTs. We observed that 43% (*n* = 23) of the apical protonema cells were able to penetrate the 1-µm gap (Supplementary Fig. S7a). Furthermore, septa (boundaries created between dividing cells) as well as many chloroplasts were observed after the gap, suggesting that the nucleus had also penetrated the small gap and had undergone cell division. On the other hand, when the gap length was extended to 100 µm, none of the cells (*n* = 49) succeeded in completely crossing the gap, even after three weeks of culturing (Supplementary Fig. S7b). In addition, cell division events were not observed in the extended gap channels.

## Discussion

The deformability of tip-growing plant cells has often been studied using PTs as a model system^3^. While the PT is covered by a rigid cell wall, the tip region is relatively flexible, enabling the tube to penetrate through the transmitting tissues on its way to the ovule^20^. In the current study, we demonstrated that, in addition to PTs, other tip-growing plant cells, i.e., root hairs and moss protonema, are also capable of becoming deformed and passing through the physical barriers in their growth paths. Nevertheless, when the cell body was constricted in the extended narrow channels, most PTs ceased to elongate or frequently burst in the middle of the channels. While the investigation of the mechanism responsible for this cause is beyond the current study, one possible reason will be that in such an extremely confined space, the continuous transport of vesicles to distant apical region would be more challenging. Since vesicles carry the cell wall component materials, impairment of their transport may disrupt cell wall construction, causing tip growth termination or bursting cells.

In contrast, the moss protonemata were not only able to penetrate through the narrow channels, but they also created multiple septa despite their highly constricted environment (channel height: 10 µm, width: 5 µm). However, when the width of the channels was reduced to 1 µm for an extended distance (100 µm), septa formation was not observed. These results suggest that the mechanisms responsible for cell division might be affected when the amount of constriction experienced by the cell body exceeds a certain limit. It is plausible that the cytoskeletal elements, such as actin filaments and microtubules, play pivotal roles in these mechanism, and the dynamics of these cytoskeletons will be analyzed in future studies.

In addition to investigating the penetration capability of these tip-growing cells, we also examined the position of nuclei in PTs and root hairs after their apical region had crossed the microgaps. The nucleus is typically located at a fixed distance from the apex of the growing root hair, which appears to be important for proper tip growth^21, 22^; Ketelaar *et al*.^21^ reported that tip growth in Arabidopsis root hairs was arrested when the nucleus was trapped in a fixed position using optical tweezers. In the current study, the root hair nucleus never became trapped at the microgap; moreover, we did not observe any unusual tip-growth behavior after the tip had crossed the gap. The capability of a root hair nucleus to penetrate through an extremely small space appears to be critical for root hair tip growth in such tight surroundings. On the other hand, for PTs, the relationship between the location of the VN and tube growth remains largely unknown. While the VN was able to penetrate through the gaps in the current study, some VNs became temporally trapped (for a maximum of approximately 8 min) at the microgaps. The PT tip still grew at a normal rate (~22 µm·min^−1^) while the VN was stationary; thus, the importance of the VN location on PT growth remains unclear. It would also be interesting to examine whether trapping the VN at the microgap would affect the PT’s ability to navigate toward the ovule, which we will investigate in future studies. It is likely that PTs elongate through tight spaces *in vivo* before entering the ovule, such as transmitting tissues and micropyles, which is true for SCs as well. Our data suggest that the flexible shape of SCs appears to be critical in helping them realize successful fertilization.

In summary, we investigated the penetration capability of tip-growing plant cells using microfabricated PDMS gaps and channels. We found that these cells possess the ability to deform their apices and nuclei, allowing them to grow through the constricted environment. The devices developed in the present study represent novel tools for studying the mechanical properties of tip-growing cells in extremely small spaces, and will pave the way for the development of new experimental designs. For instance, Honkanen *et al*.^23^ screened mutants impaired in cell wall biosynthesis and integrity in rhizoids of the liverwort *Marchantia polymorpha* and suggested the existence of a conserved mechanism for tip growth among land plants. Our microdevices may be useful for investigating the functions of the responsible genes in such mutants through the analysis of penetration capability.

## Methods

### Fabrication of microfluidic devices

Three separate microfluidic devices were used to study the penetration capability of PTs, root hairs, and moss protonemata into extremely narrow artificial gaps. All of these devices were made of poly(dimethylsiloxane) (Sylgard 184; Dow Corning), and the microchannels were prepared via a soft lithography technique. Each mold for the PDMS devices was fabricated on a silicon wafer, which was spin-coated with negative photoresist (SU-8 3005, 3010, 3025, and 3050; Microchem Corp.). A maskless lithography system (DL-1000; Nano System Solutions, Inc.) was used to pattern the microchannel designs onto the photoresist layer. To prepare the PDMS devices, a prepolymer mixture of PDMS was poured onto each mold and degassed for 30 min. After curing for 90 min at 65 °C, the PDMS layer was peeled off of the mold, and access holes to the fluidic channels were created using punches (Harris Uni-Core). The PDMS layer and a glass bottom dish were both exposed to oxygen plasma, pressed together, and heated for an additional 30 min at 65 °C to completely seal off the microfluidic network.

### Scanning electron microscopy image (SEM) acquisition

To obtain SEM images of PDMS microchannel structures, we utilized a digital microscope (VHX-1000 with VHX-D500/D510 lens; Keyence).

### Plant materials and growth conditions

For the PT study, wild-type *Torenia fournieri* ‘blue and white’ flowers and transgenic *Torenia fournieri* ‘Crown violet’ flowers (the *RPS5Ap*::*H2B-tdTomato* line^16^) were grown at 28 °C under long-day conditions (16 h light/8 h dark). To grow PTs inside of the experimental device, a pollinated pistil of *T. fournieri* was cut, and the style (1.5 cm long) was inserted into the access hole at the center of the device (Fig. 1a,b). Then, the device was kept in an incubator at 28 °C for 5–6 h. PTs that had germinated at the stigma initially elongated through the style and eventually entered the microchannels, which were filled with growth medium for PTs.

To conduct the root hair study, seeds of *Arabidopsis thaliana* accession Columbia (Col-0) or the transgenic line *UBQ10pro::H2B-mClover* were sterilized by soaking in sterile liquid (5% [v/v] household bleach and 0.02% [v/v] Triton X-100) for 5 min. The seeds were thoroughly rinsed with autoclaved Milli-Q (Millipore Corporation) water and incubated in water for two days at 4 °C in the dark. After filling the microchannels with growth medium (0.215% [w/v] Murashige & Skoog Medium, 0.05% [w/v] MES, sucrose 1% [w/v], and agar 1% [w/v]), each seed was then transferred to the well of the microdevice and germinated vertically at 22 °C under continuous white light (Fig. 3a).

For the moss protonema study, the moss *Physcomitrella patens* strain Gransden 2004 was utilized^24^. Protonema tissues were cultured on BCDAT medium in a Petri dish under continuous white light. A small amount of protonema tissue was transferred to the moss microdevice filled with BCDATG medium (Fig. 4a) and grown for 7–9 days at 25 °C under continuous light. In the experiments using 1-µm gap channels (Supplementary Fig. S7), the incubation period was extended to three weeks. The configurations for each type of microdevice are shown in Supplementary Fig. S8.

### Microscopy of tip growth in the microdevices

For time-lapse analysis of *T. fournieri* PT growth, an inverted fluorescence microscope (IX-83; Olympus) equipped with a spinning-disk confocal system (CSU-W1; Yokogawa Electric) was used. To observe the VN and SCs of a PT labeled with H2B-tdTomato^16^, the specimen was irradiated with a 561 nm laser (Coherent) and the emitted light passed through an objective lens (LPlanSApo 10 × /0.3; Olympus) followed by a bandpass optical filter (578/105 nm), and was captured by an electron multiplying charge-coupled device digital camera (iXon3; Andor Ltd.). Both bright-field and fluorescence images were captured every 20 s using the microscopy automation software MetaMorph (Molecular Devices).

An inverted fluorescence microscope (IX-73; Olympus) was used to obtain optical images of the *A. thaliana* root hairs. Fluorescence images of the root hair nuclei were obtained by exciting the specimen using a LED lamp with a green filter (530/50 nm), passing the emitted light through a dichroic filter (570 nm), and capturing the image using a CoolSNAP HQ2 CCD camera (Photometrics).

To observe *P. patens* cultured in the microdevices, an inverted microscope (AxioObserver; Carl Zeiss) with an objective lens (C-Apochromat 40x/1.2; Carl Zeiss) was used in addition to a stereo zoom microscope (Axio Zoom. V16; Carl Zeiss) equipped with a CCD camera (AxioCam 506 color and Axiocam MRc, respectively; Carl Zeiss). The brightness and contrast of the acquired time-lapse images were subsequently adjusted for clarification using Image J software (http://rsbweb.nih.gov/ij/index.html). LPX ImageJ Plugins (LpxFilter2d - bandPassOps) were also used to reduce the image noise (https://lpixel.net/services/research/lpixel-imagej-plugins/).

## Electronic supplementary material

Supplementary Information

T. fournieri pollen tube penetrating through a 1-μm PDMS gap

A vegetative nucleus and sperm cells in T. fournieri pollen tube penetrating through a 1-μm PDMS gap
