# Supplementary material for: Capability of tip-growing plant cells to penetrate into extremely narrow gaps
Source: Sci Rep. 2017 May 3;7:1403. doi: 10.1038/s41598-017-01610-w (PMC5431147; doi:10.1038/s41598-017-01610-w)
Supplement: Supplementary file 1 — Supplementary Information [file 41598_2017_1610_MOESM1_ESM.pdf]

Supplementary Information

Capability of tip-growing plant cells to penetrate into extremely narrow gaps

Naoki Yanagisawa[*1,2], Nagisa Sugimoto[3], Hideyuki Arata[1,2], Tetsuya Higashiyama[1,2,3], Yoshikatsu Sato[*3]

[1]Division of Biological Science, Graduate School of Science, Nagoya University, Furo-cho, Chikusa-ku, Nagoya, Aichi 464-8602, Japan.
[2]JST ERATO Higashiyama Live-Holonics Project, Nagoya University, Furo-cho, Chikusa-ku, Nagoya, Aichi 464-8601, Japan.
[3]Institute of Transformative Bio-Molecules (ITbM), Nagoya University, Furo-cho, Chikusa-ku, Nagoya, Aichi 464-8601, Japan.

Contact information
*Corresponding author
Tel: +81-52-789-2970; Fax: +81-52-747-2970.
E-mail address:
yanagisawa.naoki@g.mbox.nagoya-u.ac.jp (N. Yanagisawa)
sato.yoshikatsu@i.mbox.nagoya-u.ac.jp (Y. Sato)

Supplementary Movie 1. ***T. fournieri*** **pollen tube penetrating through a 1-µm PDMS gap.** Time lapse images were obtained using an inverted microscope (IX-83; Olympus) equipped with a spinning-disk confocal system (CSU-W1; Yokogawa Electric), an electron multiplying charge-coupled device digital camera (iXon3; Andor Ltd.), and objective lens (UPLFLN 60×; Olympus). The images were acquired every 10 s using microscopy automation software MetaMorph (Molecular Devices).

Supplementary Movie 2. A vegetative nucleus and sperm cells in ***T. fournieri*** **pollen tube penetrating through a 1-µm PDMS gap.** Position of the microgap is indicated by an arrow.

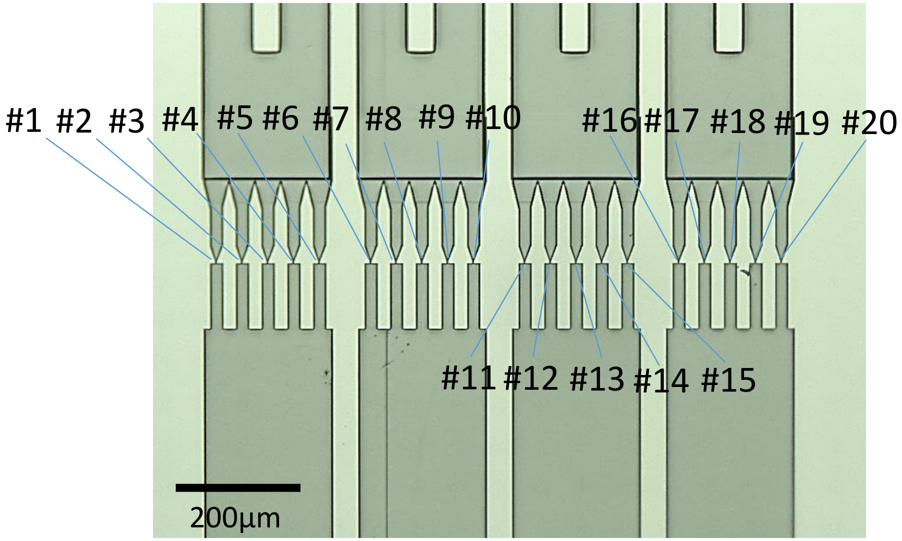

SU-8 /silicon mold used to prepare PDMS microgaps (1-µm in width) for *T. fournieri* pollen tubes

Figure S1. 1-µm wide microgap measurements. The width of the fabricated PDMS microgaps was determined from scanning electron microscopy images.

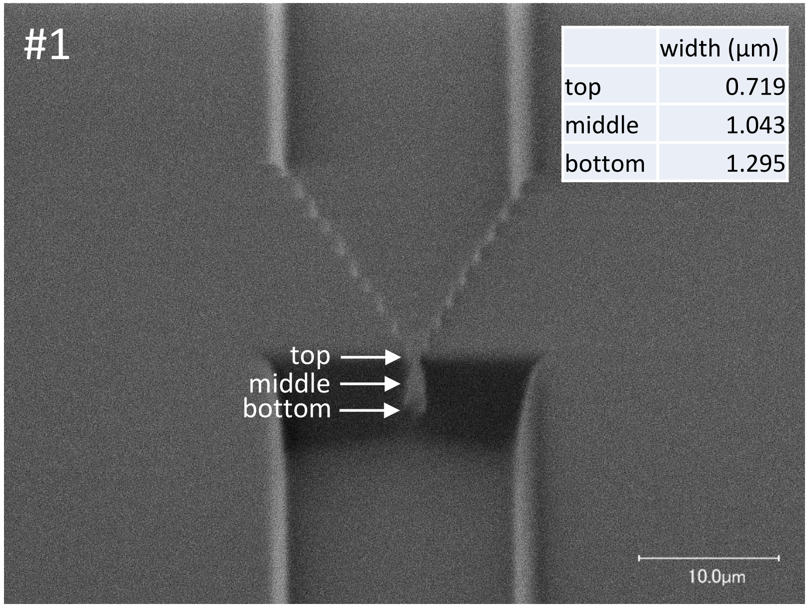

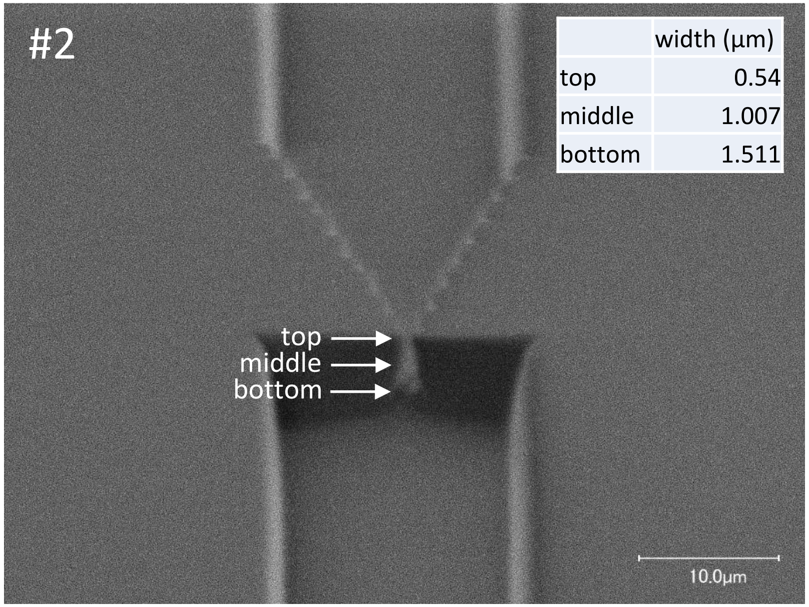

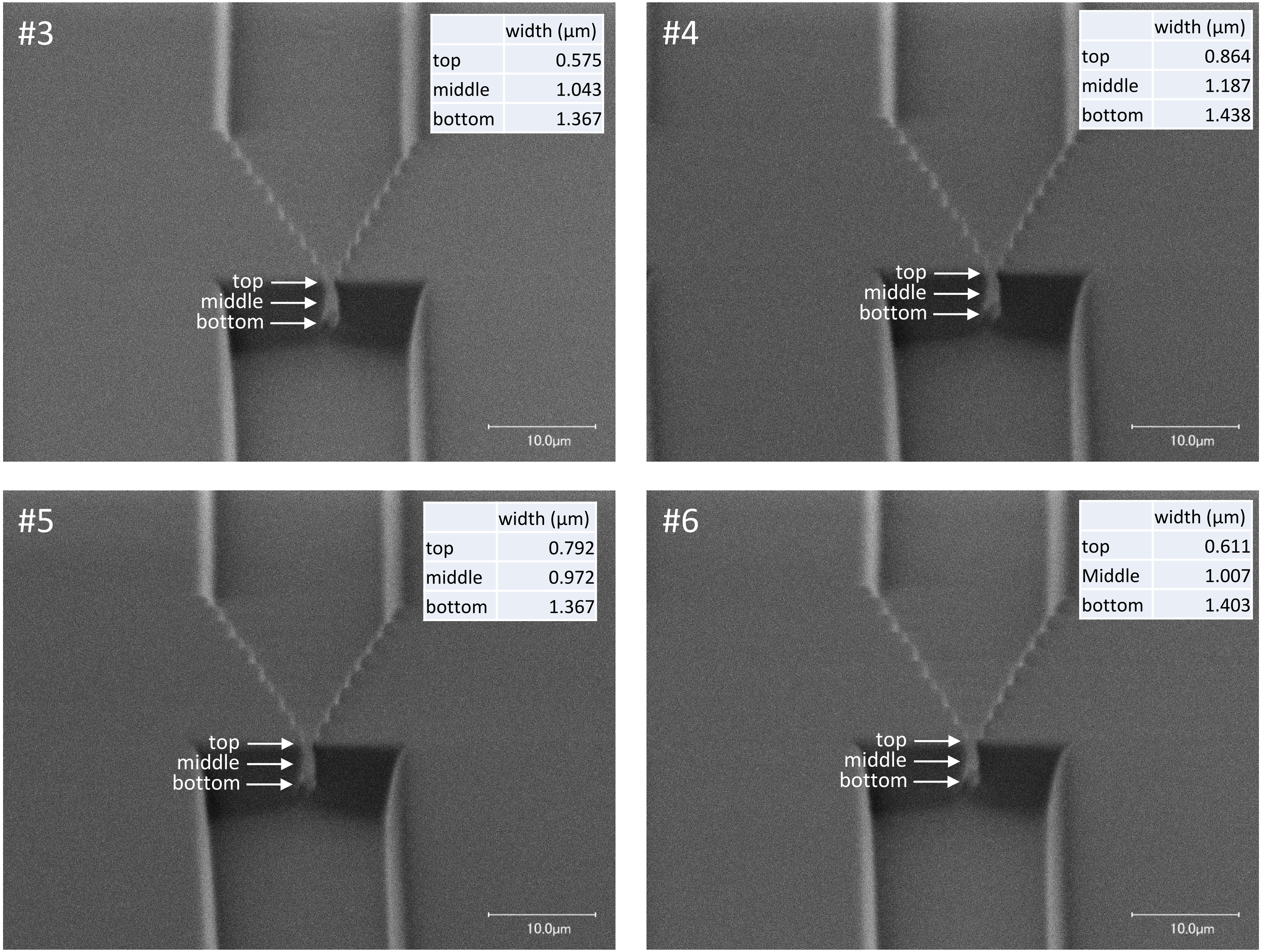
#3
width (µm)
top 0.575
middle 1.043
bottom 1.367
top
middle
bottom
10.0µm
#4
width (µm)
top 0.864
middle 1.187
bottom 1.438
top
middle
bottom
10.0µm
#5
width (µm)
top 0.792
middle 0.972
bottom 1.367
top
middle
bottom
10.0µm
#6
width (µm)
top 0.611
Middle 1.007
bottom 1.403
top
middle
bottom
10.0µm

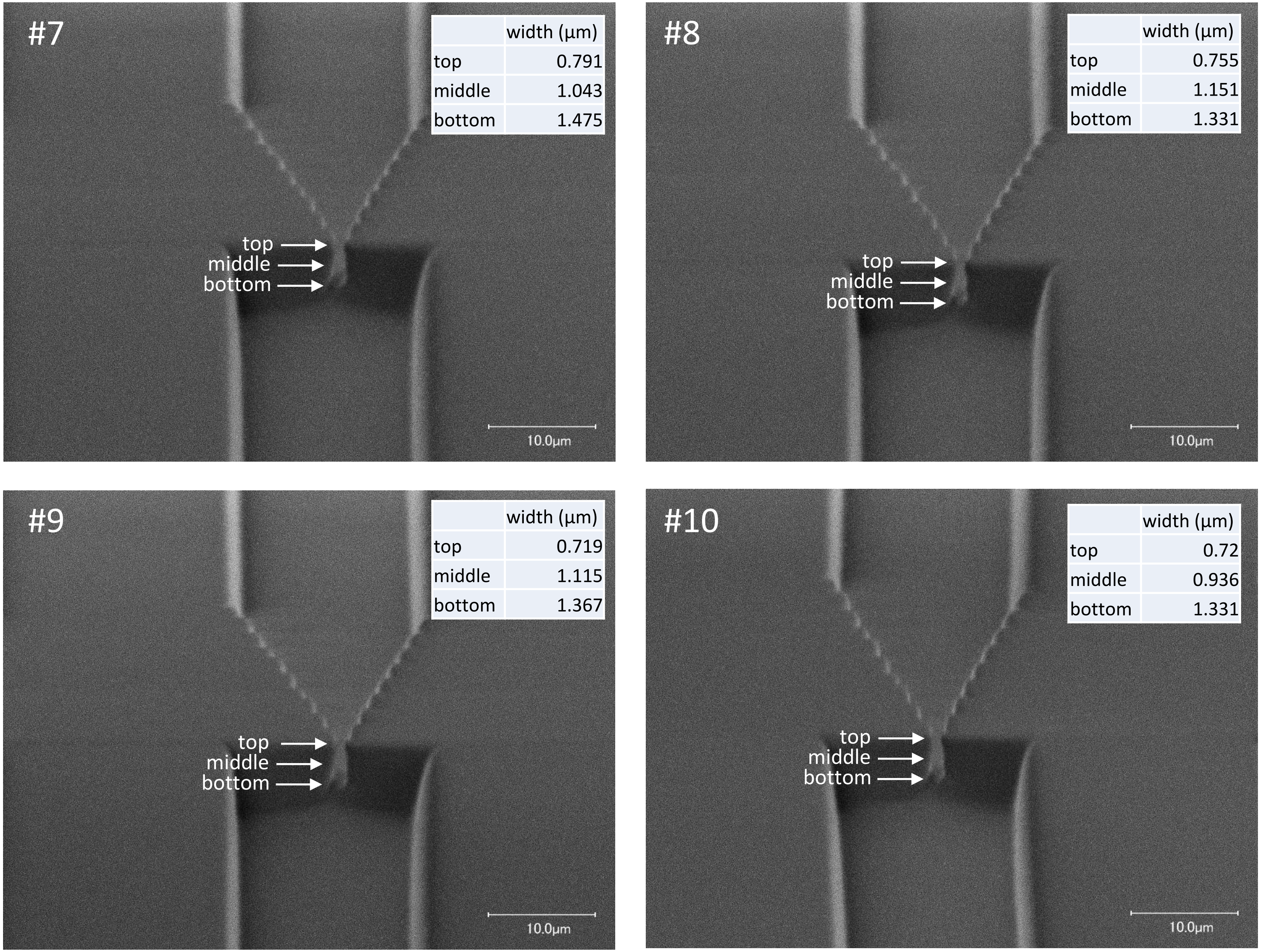
#7
width (µm)
top 0.791
middle 1.043
bottom 1.475
top
middle
bottom
10.0µm
#8
width (µm)
top 0.755
middle 1.151
bottom 1.331
top
middle
bottom
10.0µm
#9
width (µm)
top 0.719
middle 1.115
bottom 1.367
top
middle
bottom
10.0µm
#10
width (µm)
top 0.72
middle 0.936
bottom 1.331
top
middle
bottom
10.0µm

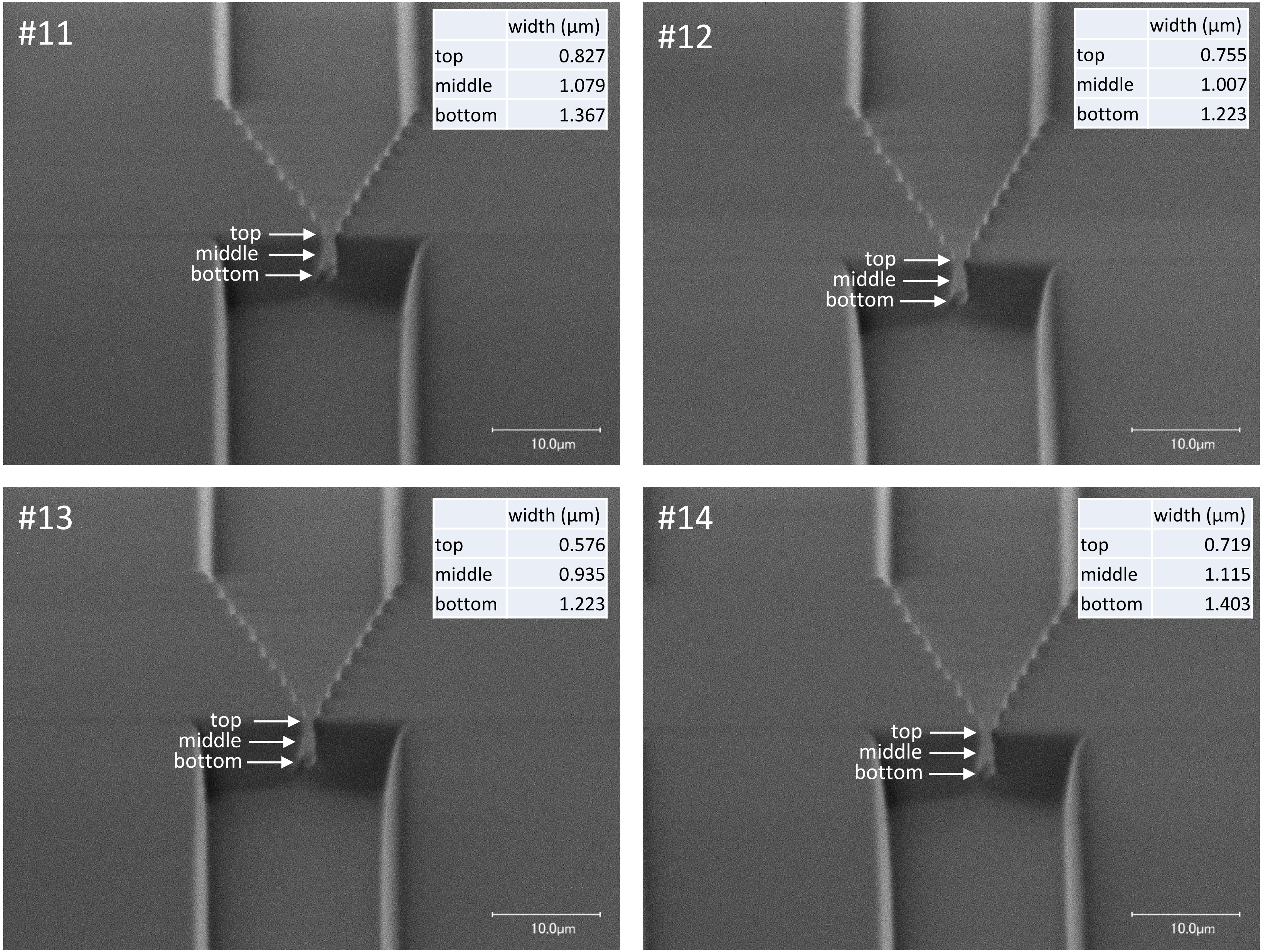
#11
width (µm)
top 0.827
middle 1.079
bottom 1.367
top
middle
bottom
10.0µm
#12
width (µm)
top 0.755
middle 1.007
bottom 1.223
top
middle
bottom
10.0µm
#13
width (µm)
top 0.576
middle 0.935
bottom 1.223
top
middle
bottom
10.0µm
#14
width (µm)
top 0.719
middle 1.115
bottom 1.403
top
middle
bottom
10.0µm

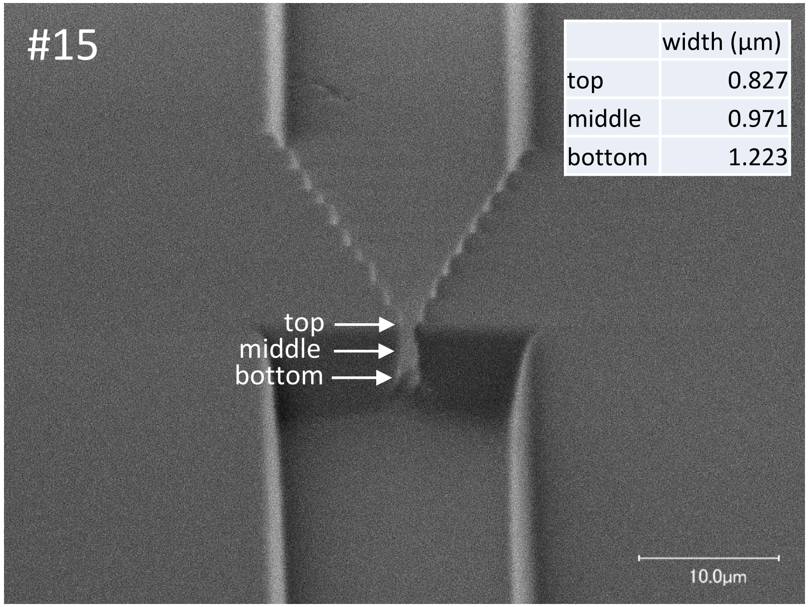
#15
width (µm)
top 0.827
middle 0.971
bottom 1.223
top
middle
bottom
10.0µm

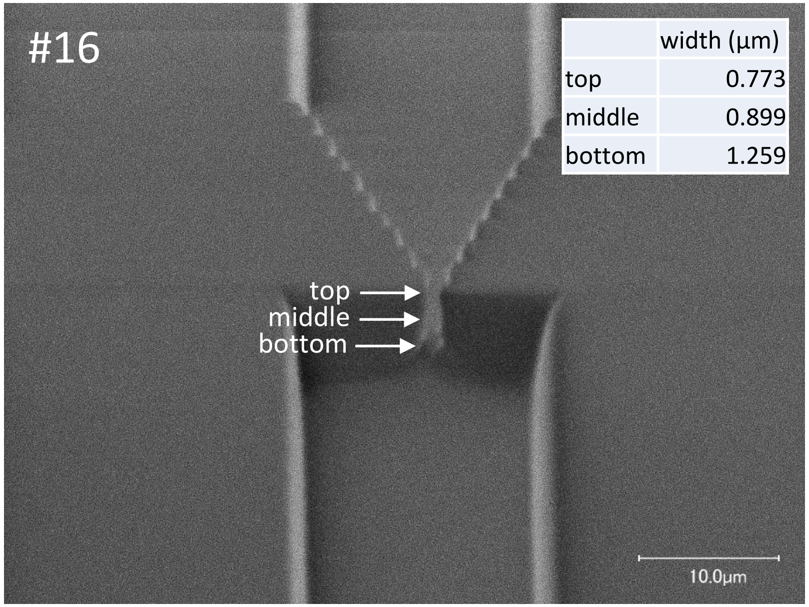
#16
width (µm)
top 0.773
middle 0.899
bottom 1.259
top
middle
bottom
10.0µm

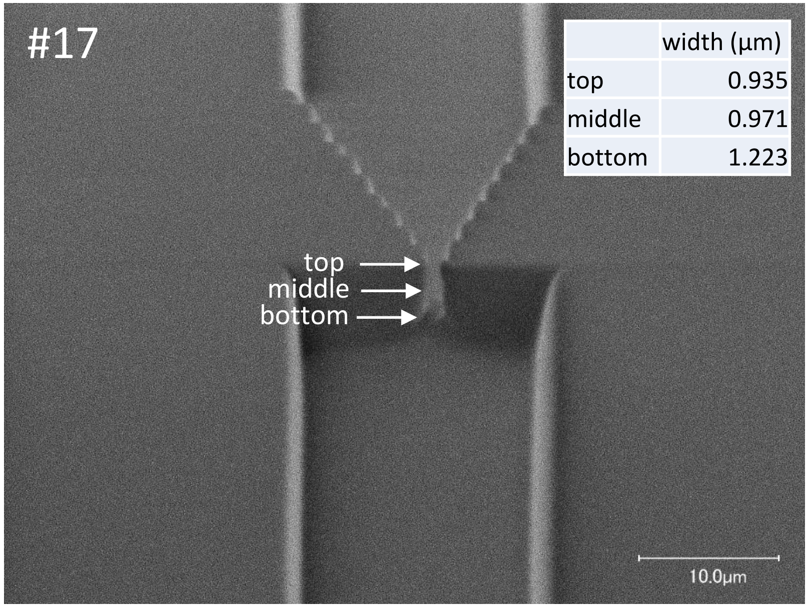
#17
width (µm)
top 0.935
middle 0.971
bottom 1.223
top
middle
bottom
10.0µm

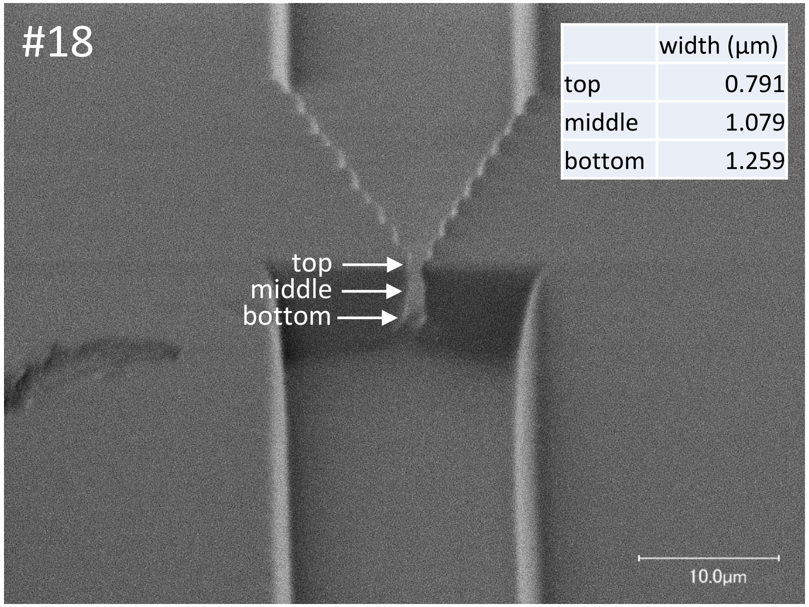
#18
width (µm)
top 0.791
middle 1.079
bottom 1.259
top
middle
bottom
10.0µm

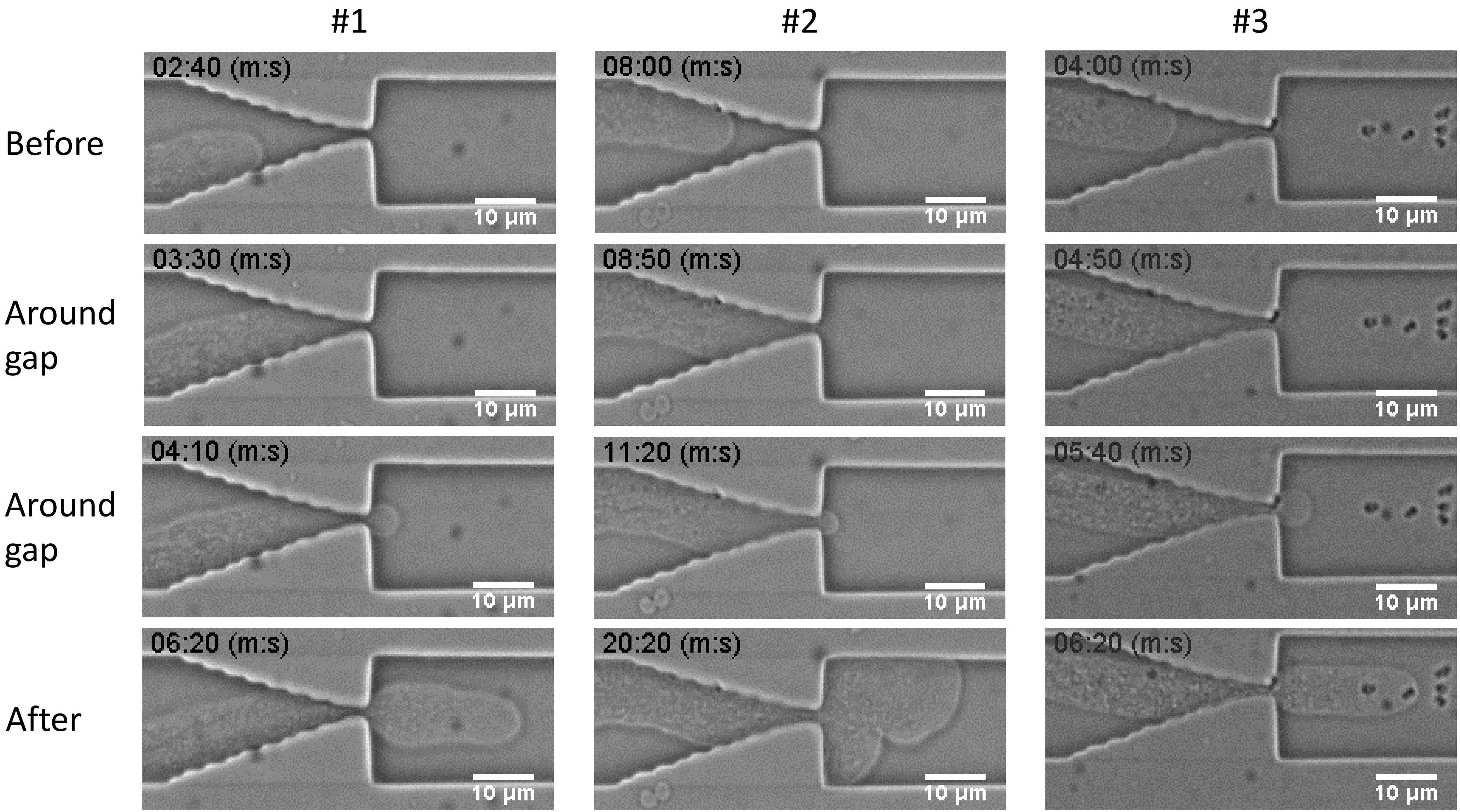

#1	around gap		after gap
time (m:s)	3:30	4:10	6:20
increment	0%	0%	0%

#2	around gap		after gap
time (m:s)	8:50	11:20	20:20
increment	0%	50%	50%

#3	around gap		after gap
time (m:s)	4:50	5:40	6:20
increment	20%	20%	0%

Figure S2. Microgap measurements during PT elongation. The degree of gap widening due to PT's turgor pressure was examined using 1-μm wide gaps (4 μm in height).

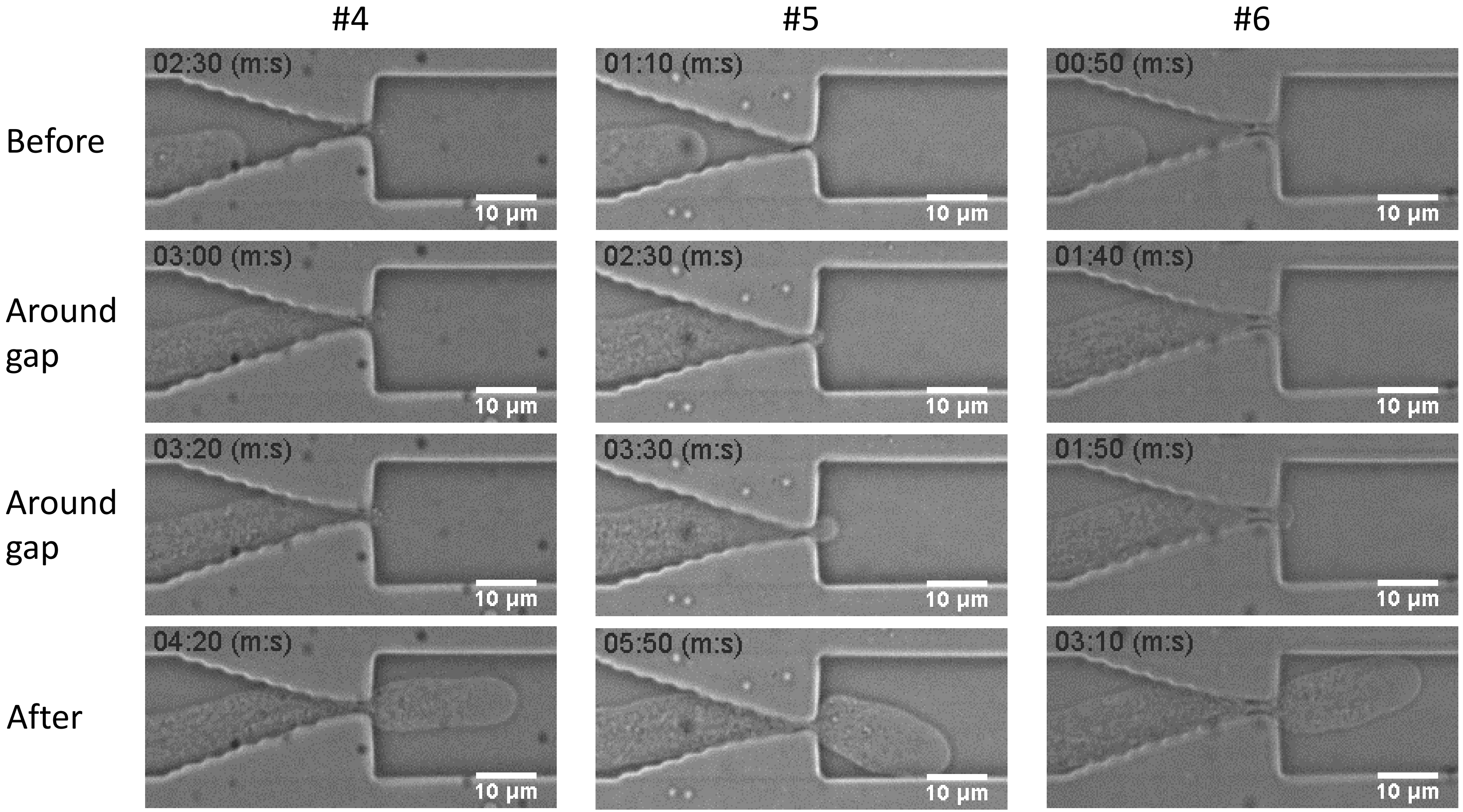

#4	around gap		after gap
time (m:s)	3:00	3:20	4:20
increment	0%	0%	0%

#5	around gap		after gap
time (m:s)	2:30	3:30	5:50
increment	25%	25%	50%

#6	around gap		after gap
time (m:s)	1:40	1:50	3:10
increment	28%	34%	62%

#7 #8 #9

Before

Around gap

Around gap

After

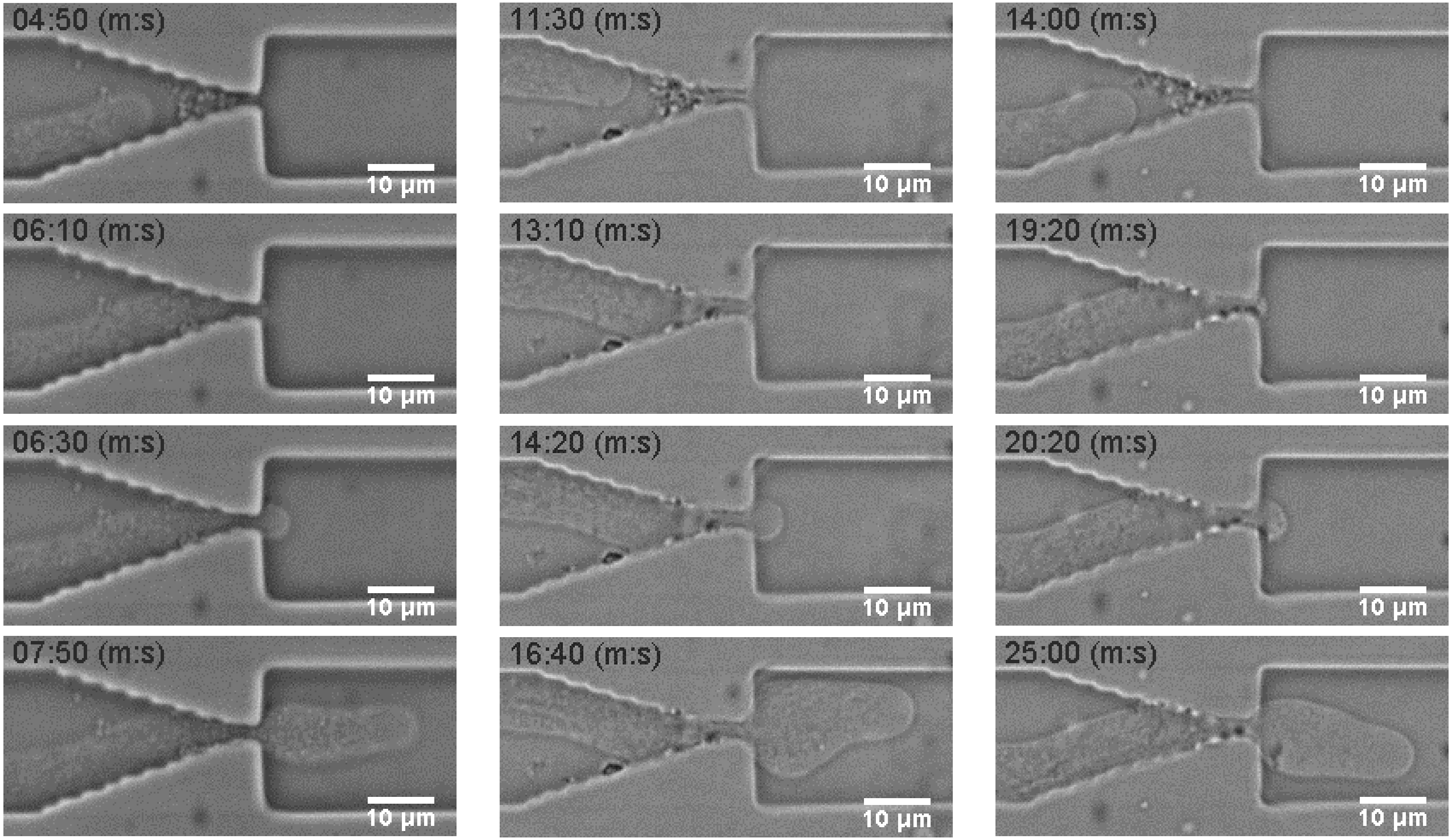

#7	around gap		after gap
time (m:s)	6:10	6:30	7:50
increment	9%	20%	40%

#8	around gap		after gap
time (m:s)	13:10	14:20	16:40
increment	29%	29%	29%

#9	around gap		after gap
time (m:s)	19:20	20:20	25:00
increment	0%	32%	29%

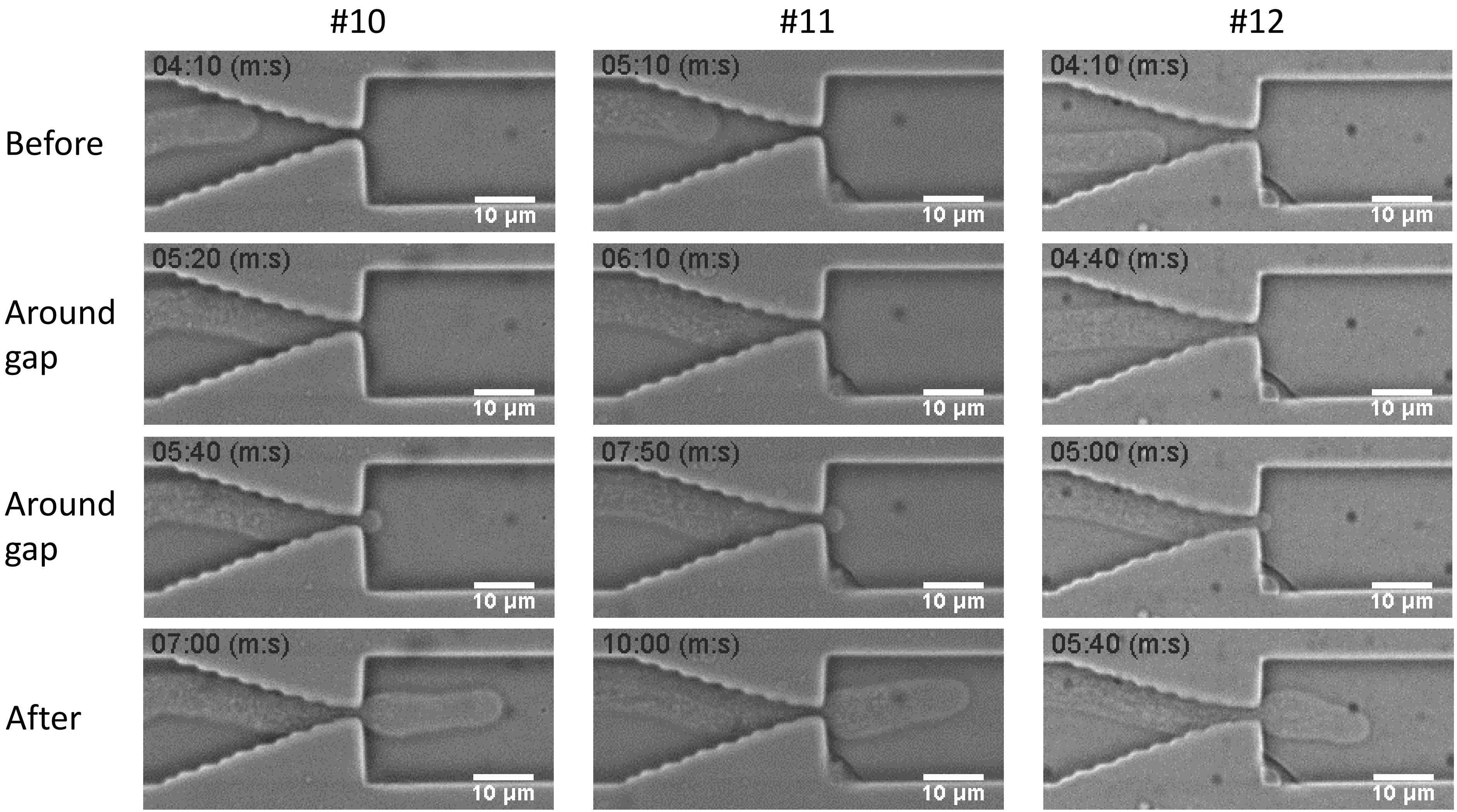

#10	around gap		after gap
time (m:s)	5:20	5:40	7:00
increment	2%	0%	0%

#11	around gap		after gap
time (m:s)	6:10	7:50	10:00
increment	0%	0%	0%

#12	around gap		after gap
time (m:s)	4:40	5:00	5:40
increment	11%	11%	27%

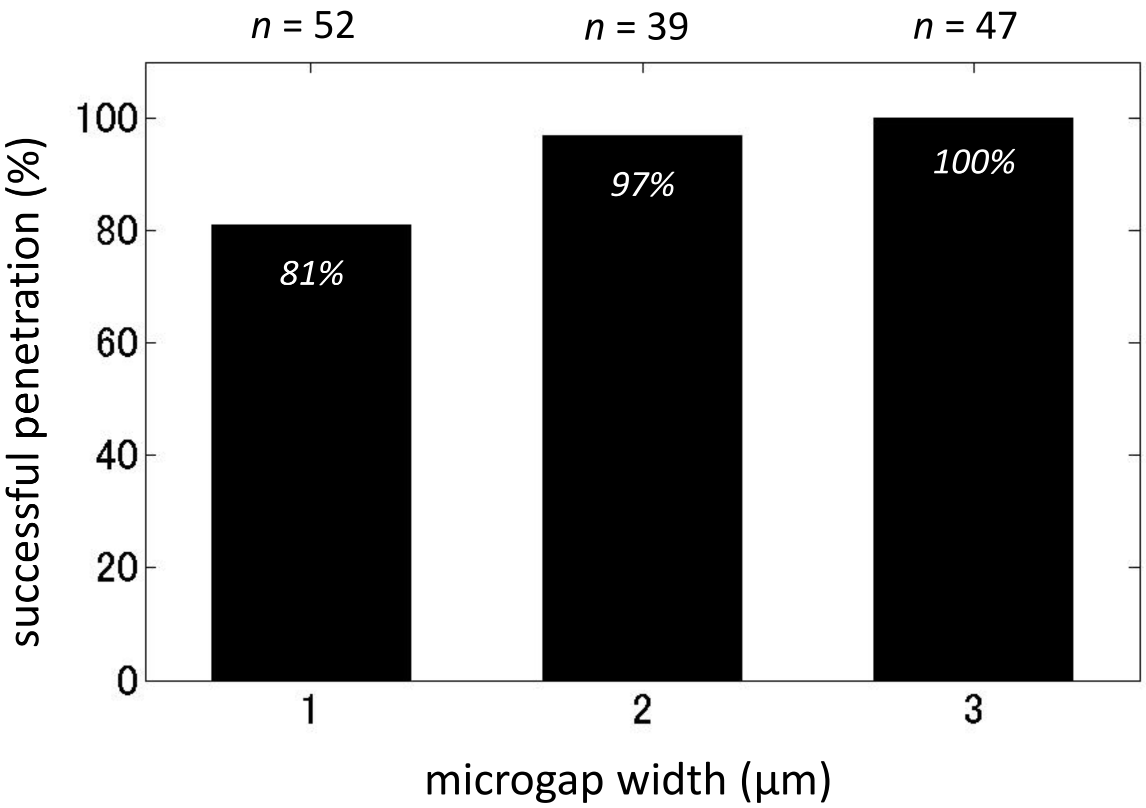

Figure S3. Capability of *T. fournieri* pollen tubes to penetrate through microgaps. 1~3 µm wide gaps (4 µm in height) were tested.

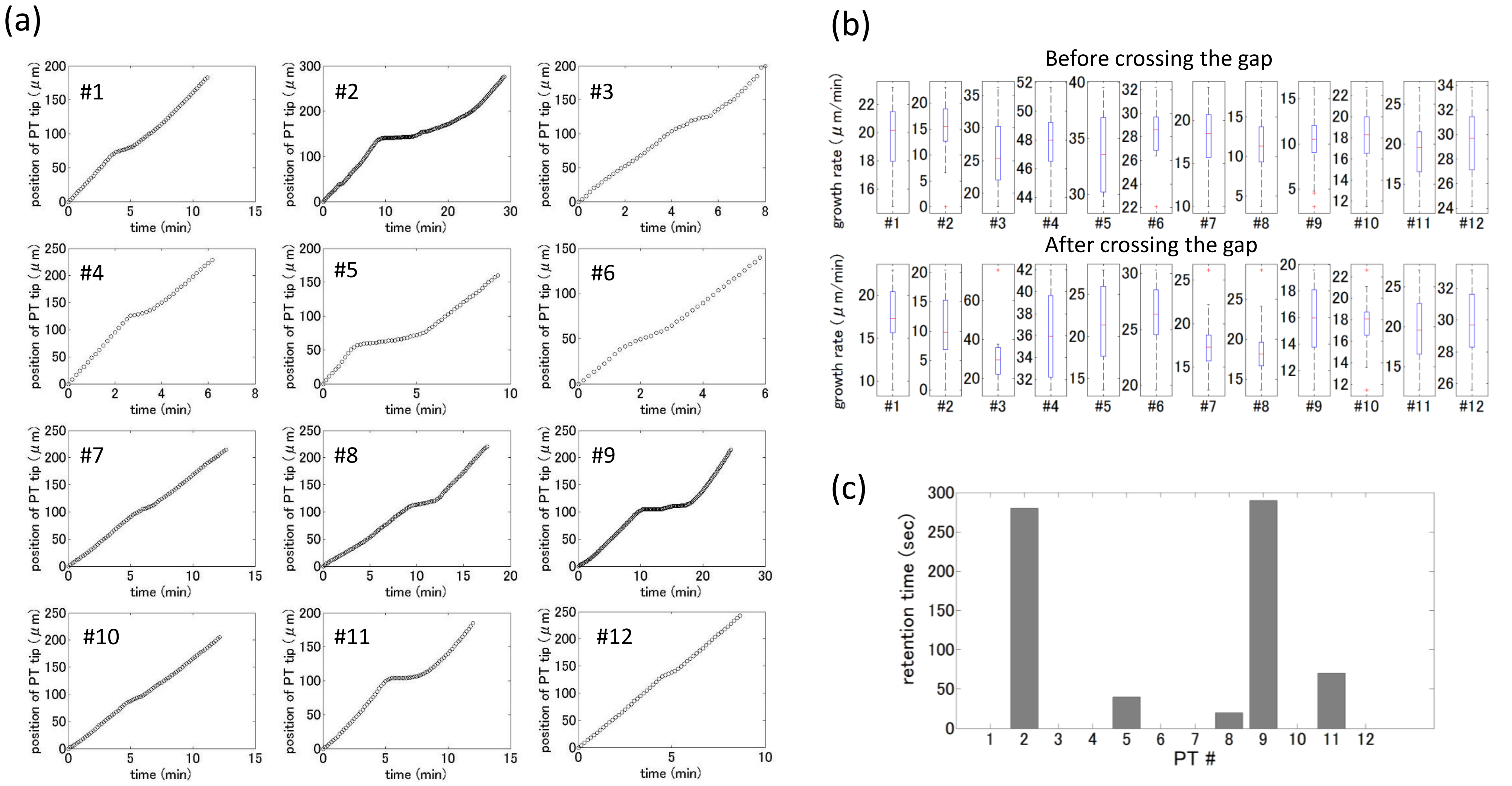

Figure S4. *T. fournieri* pollen tube growth in a microchannel with a 1-μm PDMS gap. (a) Position of PT tip in the microchannel. Time lapse images were captured every 10 sec. (b) Box plots of PT growth rate before (excluding 15 μm ahead of the gap) and after (excluding 15 μm behind the gap) crossing the gap. Outliers are set when the data points are outside of 1.5 × IQR (interquartile range). (c) PT retention time at the gap region (within up to 15 μm behind the gap). Retention of PT growth was determined when its tip position remained unchanged (i.e., growth rate = 0 μm/min in Fig S4 (a))

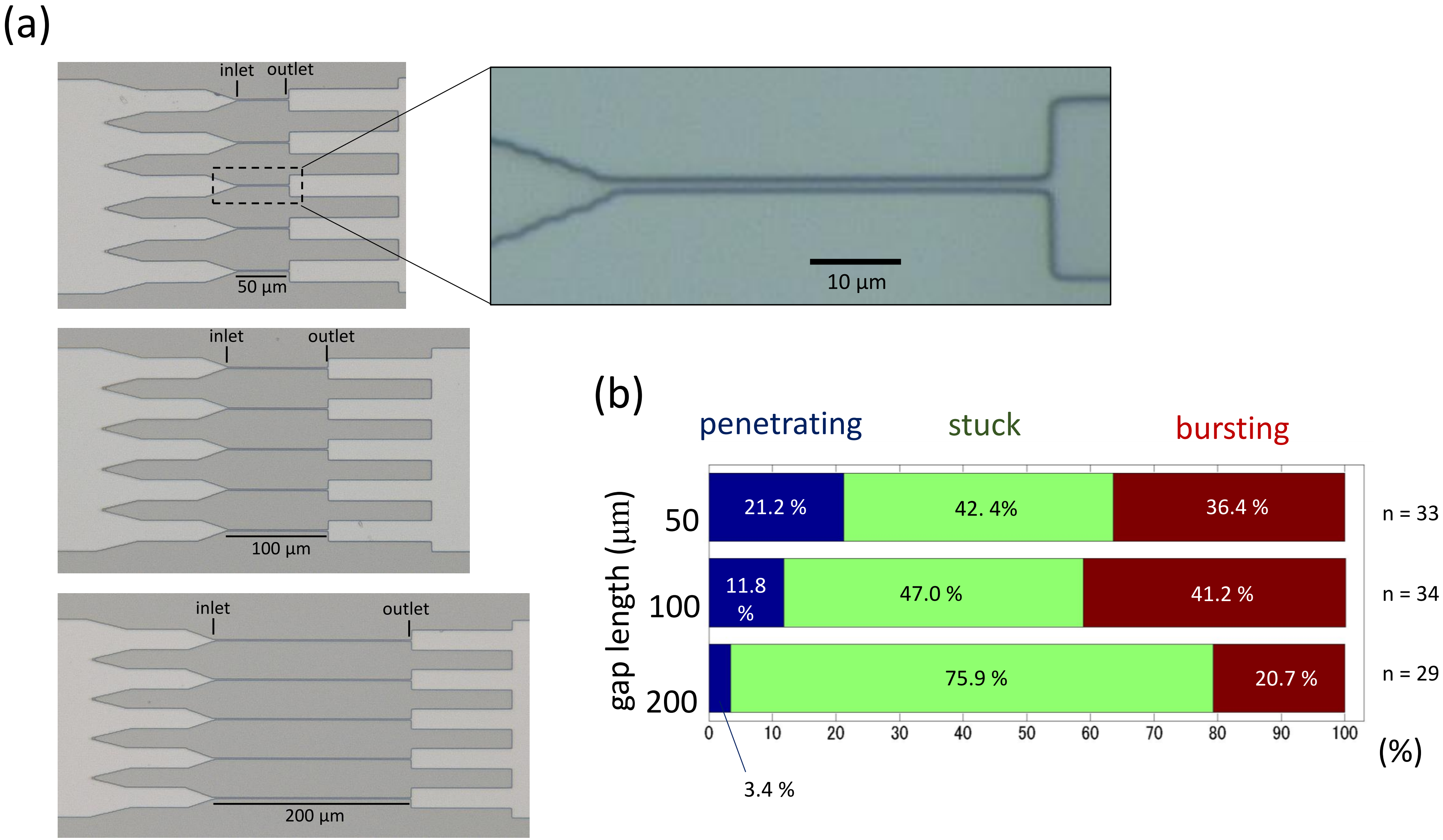

Figure S5. Capability of *T. fournieri* pollen tubes to penetrate through long gap channels. (a) Optical images of long gap channels (50, 100, and 200 µm) with a fixed width (1 µm) and height (4 µm) **(b)** Outcome of the pollen tube growth in the long gap channels.

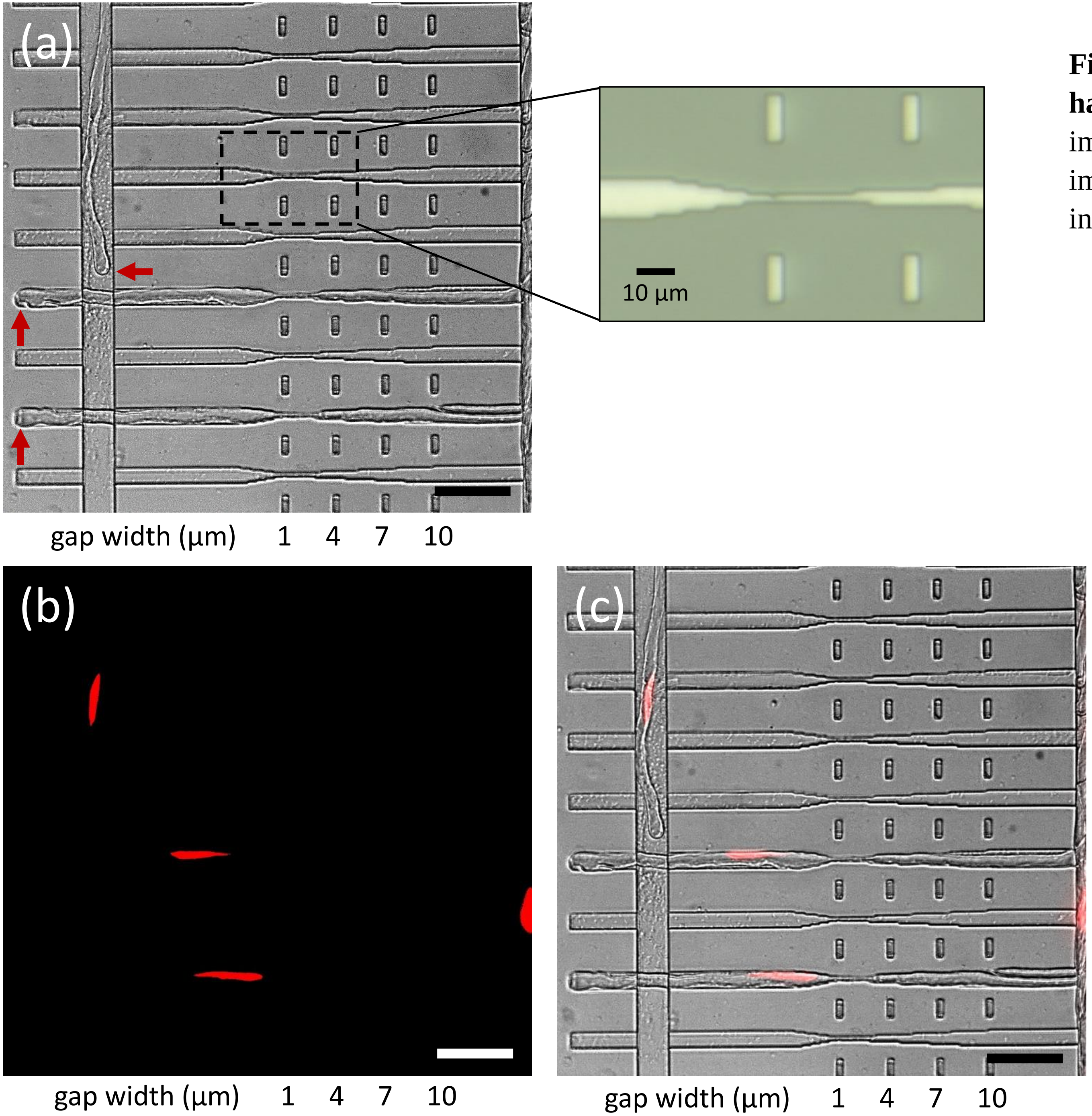

Figure S6. Nuclei of *A. thaliana* root hairs crossing 1-µm gaps. (a) bright field image **(b)** fluorescent image **(c)** merged image. Positions of the root hair tips are indicated by arrows. Scale bars, 50 µm.

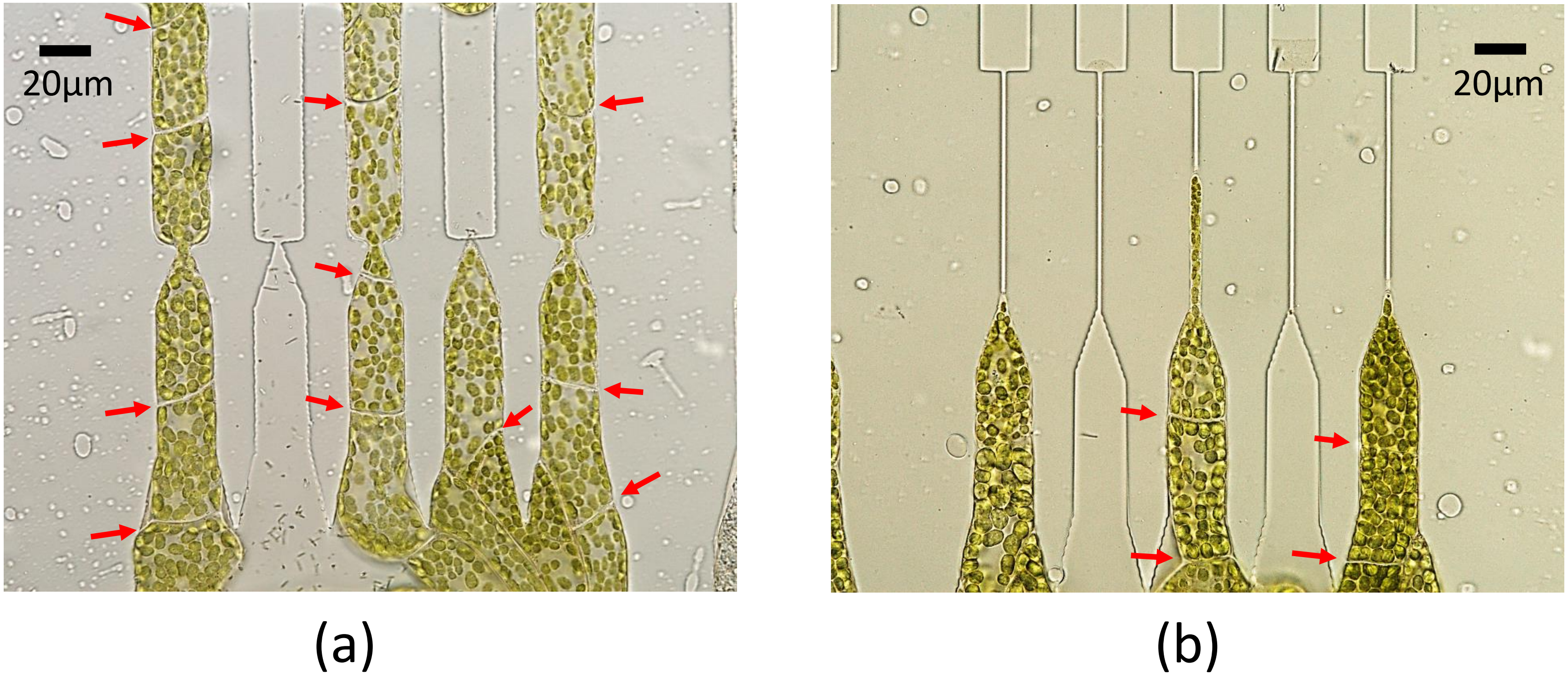

Figure S7. Study of penetration capability for moss *P. patens* protonemata. (a) Penetration of moss protonemata through a 1-µm gap. The gap was widen up to ~2 µm due to their turgor pressure. **(b)** Elongation of moss protonemata in the extended microgap (100 µm long). The protonema cells ceased their elongation in the middle of the narrow channels. Positions of the septa are indicated by arrows. Both images ((a) and (b)) were captured after 3 weeks of culturing in the microdevice.

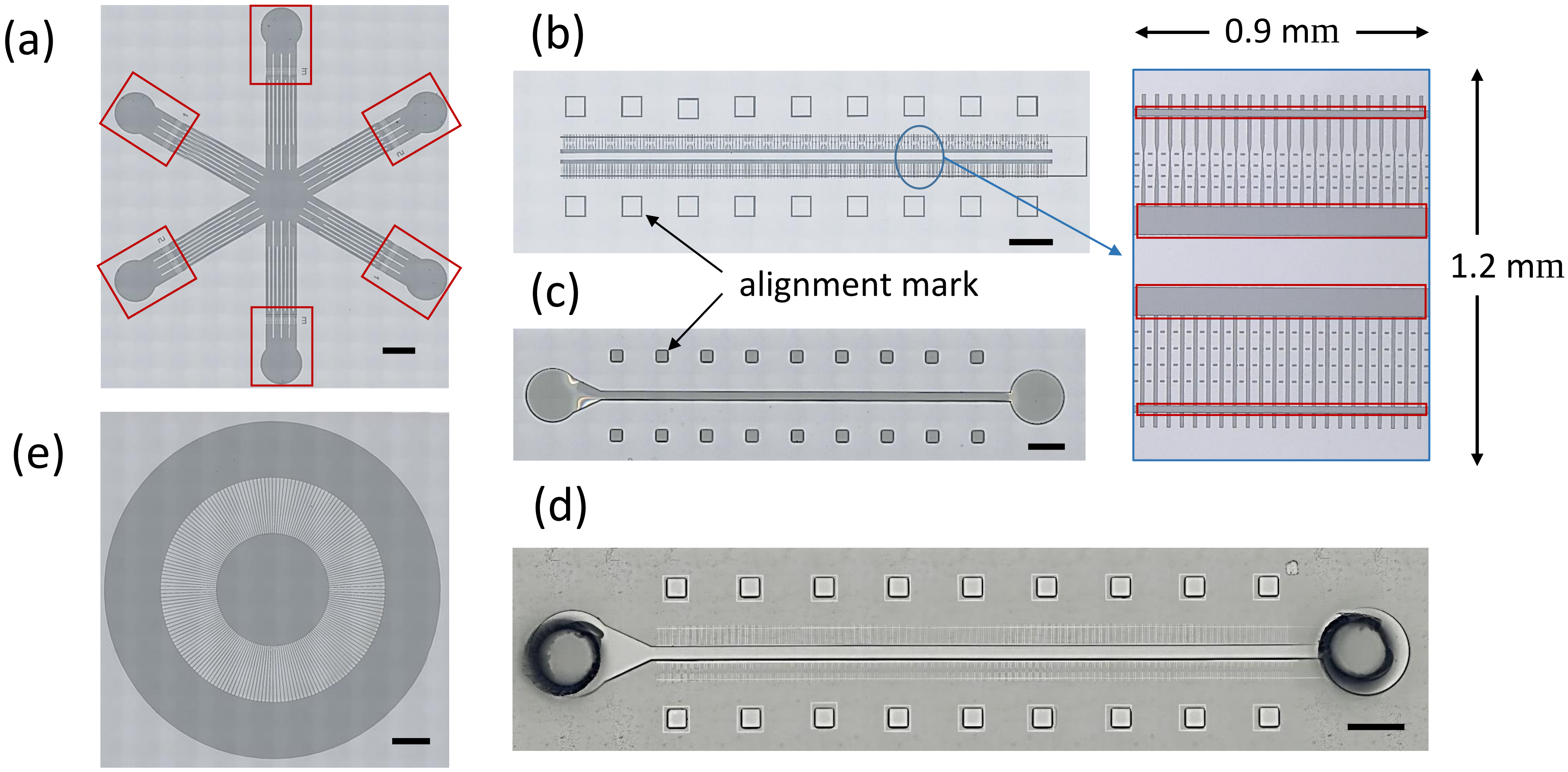

Figure S8. Microdevices employed to study the penetration capability of tip-growing plant cells. (a) Silicon mold used to prepare PDMS microdevice for *T. fournieri* pollen tubes. Channel depth, 4 µm (marked area), 11 µm (unmarked area). **(b-c)** Silicon mold used to prepare PDMS microdevice for *A. thaliana* root hairs. Microgaps are fabricated on the mold (b), channel depth, 10 µm (marked area), 4 µm (unmarked area). A microchannel used to grow a root is prepared on the mold (c), channel depth, 200 µm. **(d)** PDMS microdevice was created by assembling the PDMS layers, (b) and (c). **(e)** Silicon mold used to prepare PDMS microdevice for *P. patens* protonemata, channel depth, 10 µm. Scale bar, 1 mm.
